# Use total portosystemic shunt to rescue an emergency PNF with intractable hypotension: A case report

**DOI:** 10.1097/MD.0000000000036687

**Published:** 2024-01-05

**Authors:** Yuncong Zhang, He Dong, Xue Zhang, Juntao Wang

**Affiliations:** a Department of Anesthesiology, The First Affiliated Hospital of Shandong First Medical University & Shandong Provincial Qianfoshan Hospital, Shandong Institute of Anesthesia and Respiratory Critical Medicine, Jinan, China; b Department of Anesthesiology, The Affiliated Hospital of Qingdao University, Qingdao, China.

**Keywords:** allogeneic liver transplantation, case, hypotension, primary nonfunction, total portosystemic shunt

## Abstract

**Rationale:**

Living donor allogeneic liver transplantation is a surgical treatment for patients with end-stage liver disease, wherein a healthy liver is implanted in the patient, facilitating the recovery of the liver function in patients with end-stage liver disease. However, primary nonfunction (PNF) may occur as a result of this procedure.

**Patient concerns:**

A case of an 65-year-old Asian male with a medical history of cirrhosis and hepatocellular carcinoma is described. Intractable hypotension occurred after open hepatic portal anastomosis, and large doses of vasoactive substances did not improve the condition.

**Diagnosis:**

PNF was diagnosed during surgery and it caused intractable hypotension.

**Interventions:**

we promptly used the total portosystemic shunt to achieve a successful rescue.

**Outcomes:**

The strengthening of perioperative management and active treatment allowed second liver transplantation and anhepatic phase of up to 10 hours, following which the patient was rescued.

**Lessons:**

The lesson we have learned is that total portosystemic shunt composited with careful anesthesia management can rescue the event of PNF with intractable hypotension in liver transplantation surgery. At the same time, we give attention to blood pressure, electrocardiogram, albumin, calcium, potassium, acidosis, coagulopathy, anti-infection, and protection of vital organs is essential for successful retransplant outcomes.

## 1. Introduction

Living donor allogeneic liver transplantation is a surgical treatment for patients with end-stage liver disease, wherein a healthy liver is implanted in the patient, facilitating the recovery of the liver function in patients with end-stage liver disease. However, primary nonfunction (PNF) may occur as a result of this procedure. In liver transplantation, primary hepatic allograft dysfunction and severe hemodynamic instability, followed by hepatic portal vein opening, is a rare phenomenon.^[1]^ Herein, a case of an 65-year-old Asian male with a medical history of cirrhosis and hepatocellular carcinoma (HCC) is described.

## 2. Case presentation

### 2.1. Receptor patient information

The patient was a 65-year-old male, height 179 cm, weight 71 kg, and blood type A, RH+. The reason for this admission was liver tumor progression for 1 month. The patient had received comprehensive treatment for liver cancer for >2 years. Since the last general discharge, he had poor appetite, spirit, and sleep. The history of hepatitis B has been 14 years, and the virus content was negative. He suffered from hypertension for 15 years, and his maximal blood pressure was 160/100 mm Hg. Since nifedipine administration, his blood pressure was maintained at 130/90 mm Hg. He was allergic to sulfa drugs. After admission, the electrocardiogram showed normal sinus rhythm, indicating that the left ventricular diastolic dysfunction was reduced, and the left ventricular ejection fraction was 61%.

Preoperative diagnosis was primary hepatic malignancy, chronic hepatitis B, cirrhosis, and transcatheter radiofrequency ablation after radiofrequency ablation of hepatic malignancies.

The first donor patient information cannot be collected. Only the blood type is known to be type O, RH+. The surgical procedures of 2 liver transplants in Table 1.

**Table 1:** Anesthesia and surgical procedures for 2 liver transplants.

19:30 (date of surgery 21/11/2016)	The patient entered the operation room. The body temperature control blanket insulation was given. Noninvasive measurement of blood pressure was 170/90 mm Hg, heart rate was 79 beats/min, and sinus rhythm was normal.
19:45	A smooth monitoring of invasive radial artery pressure.
19:50	Intravenous morphine 5 mg, lidocaine 60 mg, midazolam 5 mg, sufentanil 20 mg, propofol 120 mg, benzene CIS cisatracurium 20 mg, pressurized oxygen mask, tracheal intubation after 2 minutes. Smooth tracheal intubation during anesthesia, the peak airway pressure was 15 cmH_2_O, bilateral pulmonary breath sounds were clear.
20:10	Smoothly implanted the 3-chamber catheter at the right jugular vein in the Trendelenburg position.
20:15	Swan-Ganz catheter was successfully implanted.
20:45	Increase the depth of anesthesia, then orthotopic liver transplantation was confirmed. The upper liver and the inferior vena cava were divided from the little omentum hole, gently separated and the first hepatic hilum was dissected, the hepatic artery and the hepatic duct were severed, the second hepatic hilum dissected, the hepatic inferior vena cava was freed, the left and right margins of the inferior vena cava were separated, the infrahepatic vena cava was freed, and the inferior vena cava was blocked. The amount of vasoactive substances and the concentration of inhaled oxygen was increased, the depth of anesthesia was decreased, the blood pressure within the controlled range was observed, and the hemodynamics was stable. The surgeon was told to excise the diseased liver. The surgeon trimmed the donor liver, the weight was 1495 g, orthotopic implantation, anastomosis suprahepatic inferior vena cava, infrahepatic vena cava, hepatic portal vein, open vena cava after anastomosis of the hepatic portal vein, hemodynamic stability, and bleeding was about 60 mL before portal vein anastomosis.
00:20 22/11/2016	Intravenous infusion of 580 mg methylprednisolone by the anesthesiologist, increased the rate of albumin infusion, and continuously injected 5% NaHCO_3_ by intravenous infusion.
0:30	The end of the anhepatic phase (52 minutes), hepatic portal vein flow open, the first liver transplantation surgery was completed (from 20:45 to next day 0:30). Liver edema, dark red, and hard texture were observed.
0:32	ECG suddenly reported an even number, T wave and T wave tip, height more than the R wave. Thus, the anesthesiologist immediately injected 0.3 g CaCl_2_ intravenously, which normalized the ECG. Blood gas analysis was performed immediately: blood potassium 5.5 mmol/L, blood calcium 0.8 mmol/L, Lac 6.9 mmol/L. Appropriate acid, diuretic, and dilation treatment were given: 1 g intravenous injection of CaCl_2_, intravenous infusion of 5% NaHCO_3_ in a 150 mL injection, and furosemide to promote urination. Activated clotting time of whole blood (ACT) was 185 seconds.
00:57	The blood pressure dropped from 115/70 mm Hg to 70/40 mm Hg and the heart rate increased from 90 to 120 beats/min. Thus, active treatment, continuous intravenous injection of epinephrine (5 mg/mL), norepinephrine (10 mg/mL), norepinephrine from 10 to 50 mg/kg/h continuously, dopamine from 3 to 18 mg/kg/h, and intravenous infusion of 5% NaHCO_3_ injection was administered immediately and continuously. Then, the depth of anesthesia was reduced, and the speed of infusion accelerated, followed by proper diuresis.
01:50	The hepatic portal vein was blocked, and almost no change was observed in blood pressure. The portal vein was opened randomly.
01:55	The blood pressure dropped to 63/55 mm Hg, giving the patient an ice cap to protect the brain cells.
03:20	“Portosystemic shunt” (TIPS) in advance
04:15	Blood pressure decreased to 90/62 mm Hg. Subsequently, for 4 hours, the blood pressure has been in a state of intractable hypotension and fluctuating within 80–90/60–65 mm Hg, not rising with a heart rate of 120 beats/min. Thus, we administered an intravenous injection of epinephrine 20 U and norepinephrine 30 U, and the maintenance of vasoactive substances was very high. Next, we use fresh frozen plasma and red blood cell concentration to supplement the blood volume and hemoglobin. Potassium chloride 0.3 g, calcium chloride 0.5 g, and magnesium sulfate 0.25 g were used to improve the electrolyte level, calcium chloride was given to maintain vascular tension, edema was prevented and treated with albumin, coagulation factors improved the coagulation function, ulinastatin 500,000 U was given to counteract the inflammatory mediators, 100 mL of 5% NaHCO_3_ was injected to correct acid poisoning, and urination was promoted with furosemide.
08:15	The inferior vena cava and hepatic portal vein were blocked before the second liver transplantation was begun, and the pulse pressure was reduced to < 10 mm Hg. The first liver resection and the second anhepatic phases were performed. Second donor livers were implanted, and the hepatic and inferior vena cava, inferior vena cava, and hepatic portal vein were anastomosed successively.
09:20	An intravenous bolus of 400 mg methylprednisolone was given before opening the hepatic portal vein.
09:25	They were all opened after the second new liver anastomosis, blood pressure began to rise rapidly that restored the normal blood pressure, pulse pressure, heart rate, and blood circulation. Next, we actively deepened the anesthesia, prevented reperfusion injury of vital organs, reduced myocardial oxygen consumption, used 50 mL of 5% NaHCO_3_ to correct the acidity, used 20 mg furosemide to promote urination, and improved hemoglobin and internal environment. After the opening of blood flow, the donor liver turned ruddy color and became soft in texture. After the hepatic portal vein was opened and hepatic artery anastomosed, we actively treated with heat preservation, promoted urination, acid removal, and albumin supplementation, injected fresh frozen plasma to improve coagulation function and tranexamic acid to antagonize fibrinolysis.
09:30	Urine flow was monitored. The temperature rose gradually increased from 35 to 36 °C, following which we continued heat preservation, promoted urination, corrected acid level, improved microcirculation, and provided supportive treatments based on blood gas analysis.
14:50	The end of the operation.
15:05	The patient returned to the ICU in the ward with stable vital signs. Subsequently, the first blood gas analysis was checked: pH: 7.57, Lac > 15 mmol/L, Beecf 7.3 mmol/L. Then, the ICU doctor administered active CRRT treatment.
11.24	A declining trend was observed in aminotransferase (13:30) and Lac (1 mmol/L).
11.26	The tracheal catheter was removed, the patient did not report any discomfort, and the vital signs were stable.
11.27	The patient was in good condition.

## 3. Discussion

Allogeneic liver transplantation has become a reliable approach for the treatment of irreversible end-stage liver disease. It has gained significant popularity and is beneficial in the clinical treatment of HCC. However, the complication rate of liver transplantation is increasing continuously, and the complications can be divided into 4 categories: bleeding and vascular complications, biliary complications, rejection, complications, infection complications, and PNF. Bleeding and vascular complications include massive bleeding and vascular thrombosis. Biliary complications include bile leakage, bile duct stenosis, and biliary sludge formation. The incidence of PNF is low and a severe complication of liver transplantation, which often results in graft failure and endangers recipient patient life. Shaw et al first proposed the concept of PNF, but a complete, precise, and satisfactory definition of PNF is yet lacking although it occurs in 4% to 8% of cases.^[2]^ Presently, there are some difficulties in the prediction, diagnosis, and treatment of PNF. The presence of PNF during liver transplantation and the presence of intractable hypotension are rare phenomena. Herein, we reported a case of intraoperative PNF resulting in intractable hypotension and ultimately successful rescue. In this case, the first anhepatic phase reached 10 hours, which is rare.

This case consisted of an 65-year-old Asian male with a medical history of cirrhosis and HCC, when admitted to the hospital.

The anesthesiologist attributed the reason for the drop in blood pressure dropped from 115/70 mm Hg to 70/40 mm Hg during the operation to the production of inflammatory mediators in vivo after opening hepatic portal vein and the flow back of the toxic substances by the new liver into the bloodstream, exerting great toxicity to the heart and cause circulation restraint. Occasionally, the blood pressure is difficult to maintain, requiring continuous intravenous injection of epinephrine and norepinephrine; however, the blood pressure might not increase and remains at about 80/65 mm Hg. No urine appears, and blood stasis changes in the gastrointestinal tract. It dropped from the opening of the vena cava to the opening of the hepatic portal vein, and the body temperature dropped from 36 °C to 34 °C.

**Figure 1.**
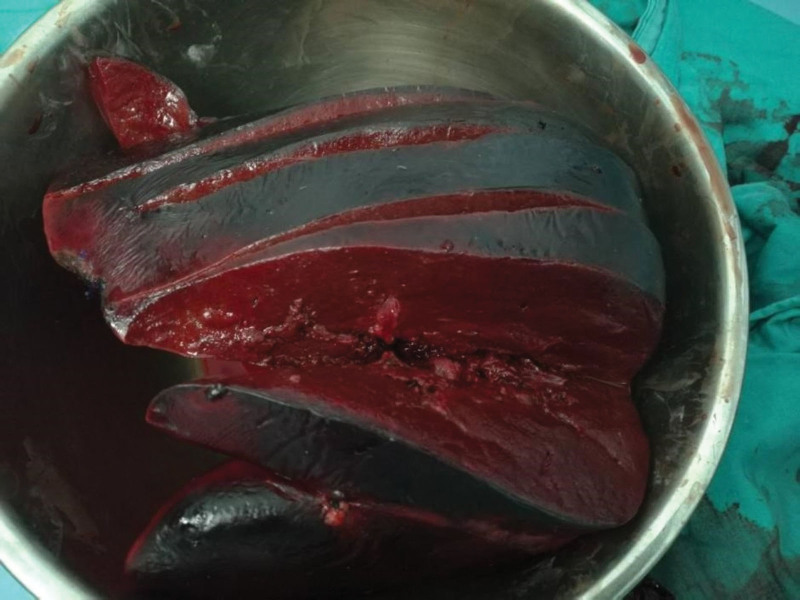
A picture of the first donor liver.

**Figure 2.**
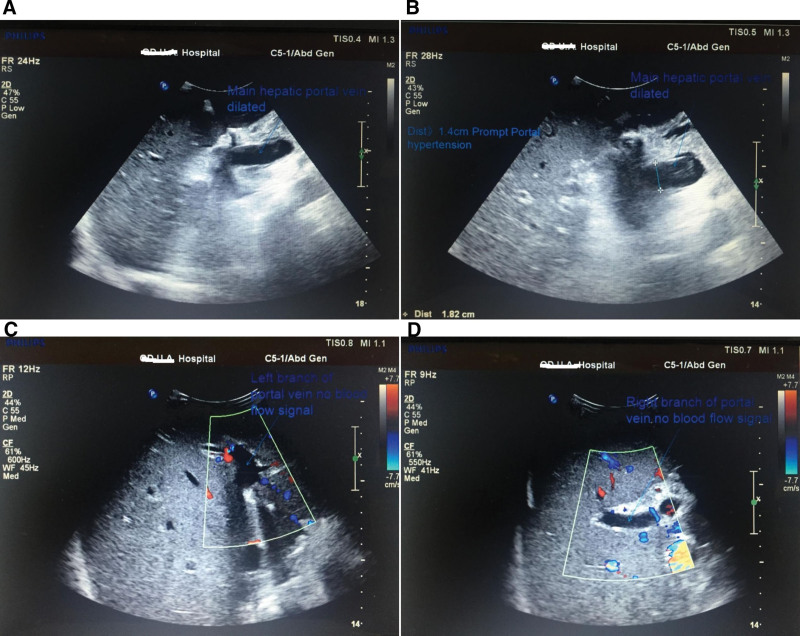
(A) Ultrasound acquisition map of hepatic portal vein dilation. (B) Ultrasound acquisition map of hepatic portal vein dilation (dist > 1.4 cm). (C) Ultrasound acquisition map of hepatic portal vein dilation (left branch of portal vein no blood flow signal). (D) Ultrasound acquisition map of hepatic portal vein dilation. (Intraoperative ultrasonography shows that the new liver was large and hard with an abnormal echo and solid change. Portal vein expansion, high tension, great resistance, and almost no flowthrough; the hepatic artery flow rate is <20 cm/s, and the hepatic vein has no blood flow.)

The anesthesiologist requested an emergency consultation with an ultrasound doctor on the operating table. Intraoperative ultrasonography showed that the new liver was large and hard, with an abnormal and uneven echo, and a solid change. The pathology post-surgery showed hepatocellular turbidity with degenerative necrosis, intrainterstitial hemorrhage, and minimal infiltration of inflammatory cells (Fig. 1), portal vein expansion (Fig. 2A and B), high tension, significant resistance, almost no flow-through, hepatic artery flow rate <20 cm/s, and no blood flow signal in the left and right branch of the portal vein (Fig. 2C and D). Comprehensive analysis has been carried out for PNF.

The anesthesiologist found it difficult to maintain blood pressure and body temperature. Thus, they suspected that the new liver may be poor quality. This serious issue was reported to the surgeon, who then contacted the artificial liver transplantation team to ensure that the heart, brain, kidney, and other vital organs were full of blood perfusion, and the patient’s quality of life had a good prognosis.

**Figure 3.**
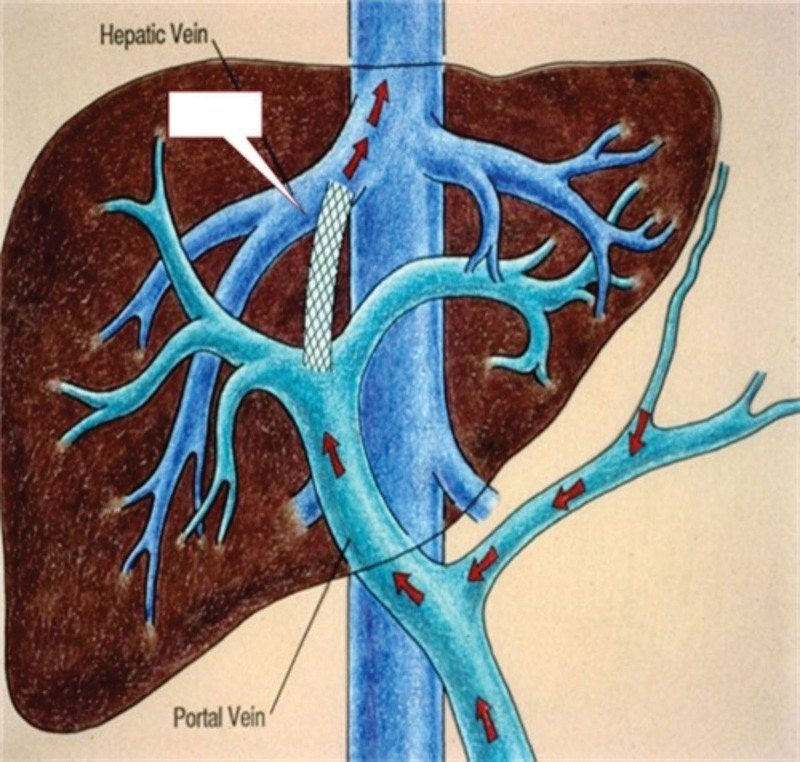
Side to side anastomosis of portal vein and infrahepatic vena cava.

The anesthesiologist stated that it was difficult to maintain the blood pressure; hence the operation plan was revised. Total portosystemic shunt (TPS) (Fig. 3) was prepared in advance, the iliac artery was corrected, the intrahepatic vena cava and hepatic portal vein anastomosed, blood pressure increased significantly 120/83 mm Hg after the opening at 03:45, and the vital signs stabilized.

Oldhafer^[3]^ speculated that liver failure is accompanied by a sharp deterioration in the cardiopulmonary function while waiting for another liver transplantation. In this urgent, life-threatening situation, transplant liver resection and temporary portal-vena cava shunt are beneficial in stabilizing the cardiopulmonary function and life-saving.

After TPS, the effective circulation volume increased, the blood volume increased, and the circulation improved, but the blood pressure began to decrease after 15 minutes.

TPS could only temporarily resolve the issue that gastrointestinal congestion cannot return, but the blood pressure is difficult to maintain. Next, we considered toxic effects from liver inflammatory mediators and other toxic substances released into the blood caused a systemic inflammatory response, thereby destabilizing the blood flow dynamics. Thus, we made an emergency contact for the new liver.

After a state of intractable hypotension for 8 hours, the second donor liver (blood type O, RH+) was prepared for transplantation.

From 23:30 on November 21 to 09:30 on November 22, the portal vein was in the blocking state and about 10 hours in the anhepatic phase, that is, 10 hours in the urine-free state.

Goal-oriented critical care management is essential to patient survival in the anhepatic phase. Anhepatic patients rapidly develop hypocalcemia, oliguria, renal failure, hypoglycemia, and hypothermia.^[4,5]^ Subsequently, the second new liver anastomosis and then opening, followed by urine flow received good feedback on the rescue efforts.

**Figure 4.**
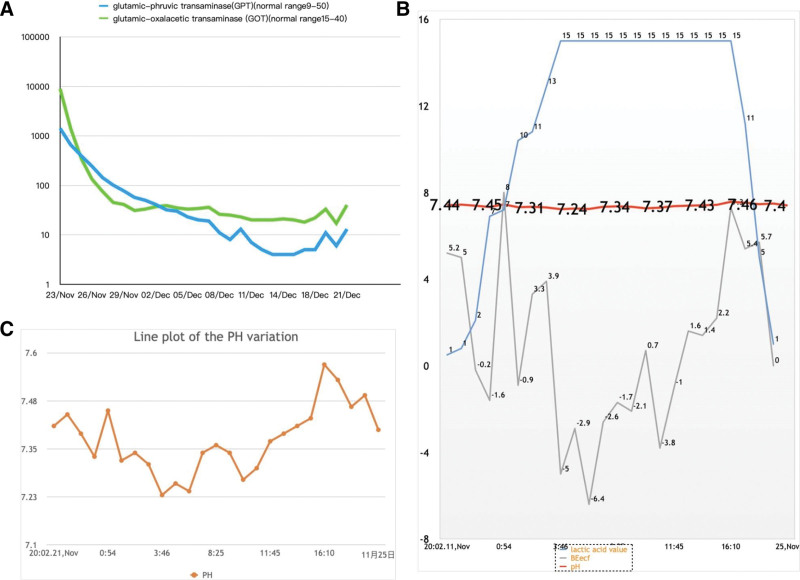
(A) Trend change of GPT and GOT. (B) Trend change of lactic acid, BE, pH. (C) Line plot of the pH variation. GOT = glutamic-oxaloacetic transaminase, GPT = glutamic-pyruvic transaminase.

The patient had stable vital signs, anastomosed biliary system, and a successfully closed abdomen after exact hemostasis. The operation was successful, and the patient was returned to the ICU ward ICU with stable vital signs. Both glutamic-pyruvic transaminase (normal range: 9–50) and glutamic-oxaloacetic transaminase (normal range: 15–40) levels normalized after 16 days (Fig. 4A), and the lactic acid, BE, and pH was improved (Fig. 4B and C).

The PNF may occur hours after transplantation and within a few days, without a clear etiology. A poor early graft function determined a complete failure of the process, and the patient died of graft failure unless they received another retransplantation.^[2]^

Singh^[2]^ summarized the characteristics of PNF as follows: acute hepatic failure (hepatic encephalopathy, ascites, coagulopathy, and hemodynamic instability), a dramatic increase in transaminase enzymes (AST and allogeneic liver transplantation, >2500 IU/L), and multiple organ failure (renal failure and pulmonary complications). Liver biopsy was helpful in the diagnosis of inflammatory cell infiltration, ballooning degeneration, and zonal necrosis of the liver cells.

The hemodynamic changes in the donor-related factors account for a large proportion. Preoperative donor hypotension, hypoxemia, and preoperative application of vasoconstrictor drugs can cause liver damage, and the recovery of liver graft function disorder. Due to the severe shortage of donors, non-heart-beating donor is increasingly used in many transplant centers.

Schemmer^[6]^ conducted animal experiments and confirmed that liver acquisition operation trauma activates Kupffer cells that can release or produce superoxide, nitric oxide, protease, tumor necrosis factor-alpha, and other cytotoxic cytokines and inflammatory factors. These lead to endothelial cell injury, platelet adhesion, and liver sinus stenosis caused by the change in microcirculation, thereby increasing the incidence of PNF. The preservation of ischemia or hypoxia injury during reperfusion and reperfusion injury during revascularization are the major causes of PNF. UW retention not only increases the effective preservation time of transplanted liver but also reduces the incidence of PNF.

Gysemans^[7]^ showed that increased endotoxin in peripheral blood during anhepatic phase also results in PNF. Endotoxin produced by severe trauma of the donor is transmitted to the recipient through the liver allograft. Intestinal ischemia and reperfusion injury could also increase endotoxin in the recipient and directly affect the function of the transplanted liver. However, the mechanism and its role in PNF need to be elucidated further.

Taut^[8]^ showed that color Doppler, hepatic arteriography, spiral computed tomography, and magnetic resonance imaging are valuable in the differential diagnosis of PNF and another early liver failure.

The most commonly accepted prognostic parameter is the activity of serum aminotransferases (glutamic-pyruvic transaminase or glutamic-oxaloacetic transaminase) (Fig. 2A), which have been utilized as comparison factors for the LiMAx test,^[9]^ and the patient recovered after the surgery.

Through this case, we shared the successful treatment experience of PNF occurrence during surgery. This would increase the clinical awareness of this currently rare operation experience, which could improve the survival rates in these patients.

(The information of this patient is obtained from the Affiliated Hospital of Qingdao University on 21/11/2016.)

## Author contributions

**Data curation:** Yuncong Zhang, He Dong, Xue Zhang, Juntao Wang.

**Methodology:** Yuncong Zhang, He Dong.

**Writing – original draft:** Yuncong Zhang, He Dong, Xue Zhang.

**Writing – review & editing:** Yuncong Zhang, He Dong, Juntao Wang.
